# A unique RET EXON 11 (G691S) polymorphism in an Indian patient with a collision tumor of the thyroid

**DOI:** 10.1186/1746-1596-2-39

**Published:** 2007-10-16

**Authors:** Bharat Rekhi, Rakesh R Badhe, Maria Alina Desouza, Devendra Chaukar, Anil K D'Cruz, Suprita Arya, S V Kane

**Affiliations:** 1 Department of Pathology, Tata Memorial Hospital, Mumbai, Maharashtra, 400012, India; 2 Tata Memorial Hospital, Department of Surgical Oncology, Tata Memorial Hospital, Mumbai, Maharashtra, 400012, India; 3 Tata Memorial Hospital, Department of Radiology, Tata Memorial Hospital, Mumbai, Maharashtra, 400012, India

## Abstract

**Background:**

Collision tumors of the thyroid are rare, with occasional reports dealing with their genetic analysis.

**Case presentation:**

A 59 year old lady presented with a neck mass, associated with hoarseness of voice of 5 years duration. Radiological examination revealed nodular masses in the left lobe of her thyroid, along with one in the isthmus, extending into the right lobe and associated with enlarged neck nodes. FNAC from the left thyroid showed features of medullary carcinoma. On total thyroidectomy, 2 distinct tumor nodules were identified in the left lobe with another in the isthmus, showing features of medullary carcinoma (MTC), papillary carcinoma and follicular variant of papillary carcinoma, respectively, accompanied with nodal metastasis. Subsequently, she underwent radioablation. *RET *gene analysis of the patient, her 2 daughters and a grandson revealed a unique G691S polymorphism on Exon 11.

**Conclusion:**

This unique case of a collision tumor of thyroid, including component of an MTC deals with the value of *RET *gene analysis and therapeutic implications in the index case and in family members.

## Introduction

The two discrete functional cellular components of a thyroid gland i.e. follicular epithelium and parafollicular C cells give rise to distinct neoplasms i.e. differentiated follicular or papillary thyroid carcinoma (PTC), from the former type and a medullary thyroid carcinoma (MTC) from the latter type of cells [1]. A MTC manifests either in sporadic or in hereditary form; the latter occurs as an isolated familial MTC or as a part of a multiple endocrine neoplasia (MEN I and II) syndrome [2]. Varying admixture of the two cell types, designated as a mixed tumor, is uncommon, but reported in a substantial number of cases [3-6]. This entity has also been recognized in the WHO classification of thyroid tumors^1^. However, occurrence of distinct tumor nodules, without an intermingling of the cell types, known as a 'collision' or a 'concurrent' thyroid tumor that represents < 1% of all thyroid malignancies, has been reported as few case reports [7-10]. Further, only occasional case reports have dealt with the genetic analysis in a collision thyroid tumor [11]. While activating germline point mutations in the *RET *gene are responsible for MTCs (associated with MEN 2), *RET *rearrangements in form of fusion of the RET cytoplasmic kinase domain to 5-ter of heterologous genes, generating the chimeric *RET/PTC *oncogenes, are linked to PTCs [12]. Herein, we describe a case of a collision tumor of the thyroid with metastatic lymph nodes. On genetic analysis, the patient revealed a unique G691S polymorphism on the exon11 of the *RET *Proto-oncogene that was also identified in both her daughters and a grandson. This case is discussed with its possible genetic and therapeutic implications.

## Case presentation

A 59 years old lady presented with the complaints of a slowly increasing thyroid swelling, since 5 years, accompanied with hoarseness of voice; dry cough for two years and an increasing pain since 6 months. She denied any symptoms of hypo- or hyperthyroidism; dysphagia, abdominal pain, irradiation in the head and neck region or any family history of thyroid cancer.

On examination, a large, firm to hard, non tender thyroid mass measuring 6 × 5 cms was noted in the infrahyoid region of the neck, more so towards the left side, associated with an enlarged left level II cervical lymph node that measured 3 × 3 cms. On laryngoscopy (Hopkin's), both the vocal cords were normal in mobility. However, the mass was presumed to be involving the strap muscles. There were no manifestations of MEN syndrome. A clinical stage T_4a_, N_1_, M_0 _for thyroid cancer was assigned. Subsequently, she underwent a fine needle aspiration cytology (FNAC) on which a diagnosis of medullary carcinoma was rendered. She underwent a total thyroidectomy with bilateral complete cervical nodal clearance. A final histopathological diagnosis of a 'collision' tumor of the thyroid, including components of MTC and PTC, occurring as discrete tumor nodules in the left thyroid, along with a FVPTC in the isthmic tumor nodule, was offered. In addition, the left cervical nodes revealed nodal metastasis of MTC.

## Radiological findings

### Ultrasonographic (USG) Neck findings

**Figure 1:**
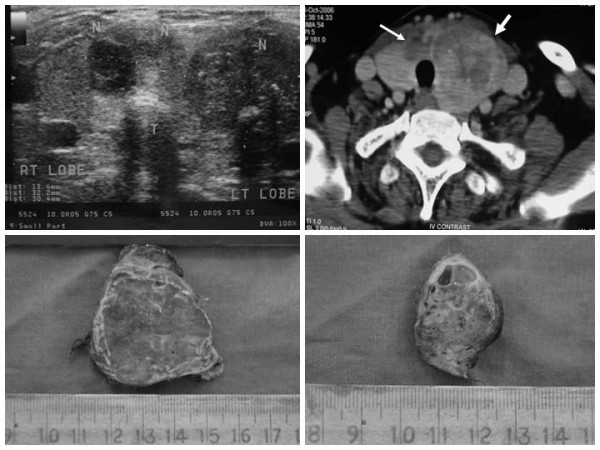
A. USG findings neck. An enlarged heterogenous mass in the left lobe of the thyroid along with another cystic hypoechoic nodule in the isthmus extending into the right lobe (thin arrow). B. CT Scan findings. an enlarged heterogeneous mass with areas of calcification and 'necrosis' in the left lobe of the thyroid (thick arrow) along with another discrete cystic nodule in the isthmus (thin arrow). C. Gross findings. A solid, fleshy, grey white tumor in the left lobe of the thyroid. D. Isthmic nodule exhibiting multiple cystic areas.

An enlarged heterogenous mass was noted in left lobe of the thyroid, measuring 6.8 × 4.4 × 3.5 cms, along with another heterogeneous hypoechoic nodule in the mid and inferior pole of the left thyroid lobe, measuring 2.6 × 2.5 × 2.1 cms. In addition, there was another hypoechoic nodule in the isthmus measuring 2.5 × 1.8 cms, extending into the right lobe and revealing cystic areas. Enlarged level II and level IV nodes were also noted. 'Specks' of calcification were identified in the thyroid mass as well as in the lymph nodes. (Figure 1A). The parathyroid glands were normal. USG findings of abdomen did not reveal any specific abnormality.

### Computed Tomography (CT) scan findings

An enlarged heterogeneous mass was noted in the left lobe of the thyroid, pushing the ipsilateral strap muscles and trachea and showing areas of calcification and 'necrosis'. The ipsilateral major neck vessels were also displaced posteriorly, along with multiple enlarged nodes at levels II and III. CT findings of thorax and abdomen were within normal limits (Figure 1B).

## Methods

The resected tumor was processed for paraffin sections. Five micron thick sections were stained with hematoxylin and eosin (H& E), alkaline Congo red stains and further subjected to immunohistochemistry (IHC), using streptavidin-biotin method. The various antibody markers performed were calcitonin (polyclonal, 1:50 dilution, Dako-CAL-3F5), thyroglobulin (monoclonal, 1:50 dilution, Dako), carcino embryogenic antigen (CEA, polyclonal, 1:600 dilution, Dako) and chromogranin (polyclonal, 1:100 dilution, Dako).

Further, the patients' blood was submitted for genetic testing for *RET *gene analysis. DNA was isolated using standard protocol [13]. Tissue DNA analysis was not performed. Direct sequencing of PCR products was used to detect the mutations in the various exons, namely 10, 11, 13, 14 and 16 of the *RET *oncogene. Rest of her family members, including her children were counseled, who also underwent the similar genetic analysis.

### Amplification and sequencing analysis

DNA extraction was done using Qiagen column and PCR protocol. Genomic DNA was amplified by PCR using primers described previously [11]. The PCR product was directly sequenced using Big Dye Terminator v3.1 cycle sequencing kit, nucleotide sequencing using AB13100 Genetic Analyzer. The sequences were analyzed with reference sequence using ClustaIW software and the polymorphisms were identified.

## Results

### Biochemical findings

Preoperatively, the serum calcitonin levels were found to be high i.e. 32,150 pg/ml. The serum levels for total and ionic calcium were low i.e. 6.4 mg/dl (Normal: 8.6–10 mg/dl) and 3 mg/dl (Normal: 4.5–5.3 mg/dl), respectively. Serum T3, T4, thyroid stimulating hormone (TSH) and parathyroid hormone (PTH) levels were within normal limits. Total and fasting blood sugar levels were normal. The 24-hour urine levels for vanyl-mandelic acid (VMA) were 2 mg/day (Normal: 15 mg/day) and for metanephrines were 91.8 μg/day (Normal: 0–350 μg/day), both within normal limits.

### Pathological findings

Grossly, a specimen of total thyroidectomy was received that measured 11 × 10 × 4 cms and consisted of right lobe, measuring 7.3 × 3 × 2.5 cms; isthmus measuring 4 × 3.5 × 2 cms and left lobe, measuring 11 × 6 × 5 cms. Externally, the capsule was intact. On serial sectioning, a solid, fleshy, grey white tumor measuring 8 × 3.5 × 2.5 cms was seen involving the left lobe of the thyroid and focally abutting the thyroid capsule. A separate, well-defined, grey-white nodule measuring 2.5 × 2.1 × 1.8 cms was also noted at the apex of the same lobe. Another ill defined nodule in close proximity to the larger nodule was seen measuring 1.5 × 1.2 × 0.8 cms and involving the isthmus and adjacent right lobe of the thyroid. This nodule exhibited multiple cystic areas containing blood and colloid with focal papillary excrescences. Extrathyroid extension was noted (Figure 1C, Figure 1D).

The left and right central compartment nodes, right level IIA, IIB, III and IV nodes and the left level IIA, III, IV and V nodes were also sampled separately, the largest measuring 3.5 × 2.3 × 1.8 cms.

### Microscopic findings

**Figure 2:**
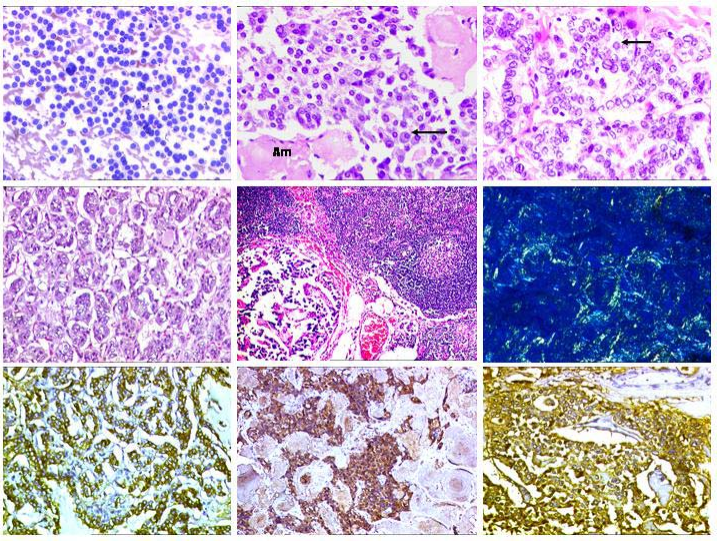
Microscopic findings. A. Cellular smears from left lobe showing tumor cells with eccentric nuclei, mostly dispersed singly with hyperchromatic nuclei. Giemsa staining 400 ×. B. Section from the left lobe nodule showing sheets of plasmacytoid cells (arrow) with 'salt and pepper-like' chromatin amidst amyloid (Am) stroma. H & E 200 ×. C. Section from second nodule showing nuclear features of PTC, including nuclear grooves and intranuclear inclusions (arrow). H & E 400 ×. D. Isthmic nodule showing features of FVPTC. H & E 200 ×. E. Nodal deposits from MTC. H & E 200 ×. F. Apple green birefringence of amyloid stroma. Alkaline Congo Red 200 ×. G. Positive thyroglobulin expression in PTC. DAB 400 ×. H. Calcitonin positivity in MTC. DAB × 200. I. Positive CEA expression in MTC. DAB × 400.

FNAC smears were hypercellular with oval to polygonal cells, singly dispersed, including binucleate and plasmacytoid forms. Cells exhibited moderate amount of granular cytoplasm and 'salt and pepper' like nuclear chromatin. Few tumor giant cell forms were also noted. A diagnosis of a medullary carcinoma thyroid was rendered (Figure 2A).

#### Histopathological findings

H& E stained sections from the left thyroid nodule showed 2 tumor nodules separated by a sleeve of normal thyroid parenchyma. The larger nodule showed features of a MTC, including sheets and nests of cells, embedded in a hyaline matrix, with plasmacytoid shape and neuroendocrine features and the other nodule, showing features of a differentiated papillary carcinoma with tumor cells, exhibiting 'optically clear' nuclei with grooves and intranuclear inclusions. Sections from the isthmic nodule showed features of follicular variant of papillary carcinoma (FVPTC). Extrathyroid extension was seen. Out of a total of 44 sampled nodes, 4 showed metastasis of MTC, two from the left central compartment and one, each, from the right central compartment and the left level IIB nodes. Perinodal extension was noted (Figure 2B, C, D, E).

Alkaline Congo red staining showed 'apple-green' birefringence of the hyalinized material, under a polarizing microscope, thereby confirming the presence of amyloid stroma, in the areas of MTC (Figure 2F)..

On immunohistochemistry (IHC), calcitonin CEA and chromogranin were found to be positive in the tumor cells of medullary carcinoma, while TG was positive in the tumor cells of PTC and FVPTC (Figure 2G, H, I).

### Genetic analysis

**Figure 3:**
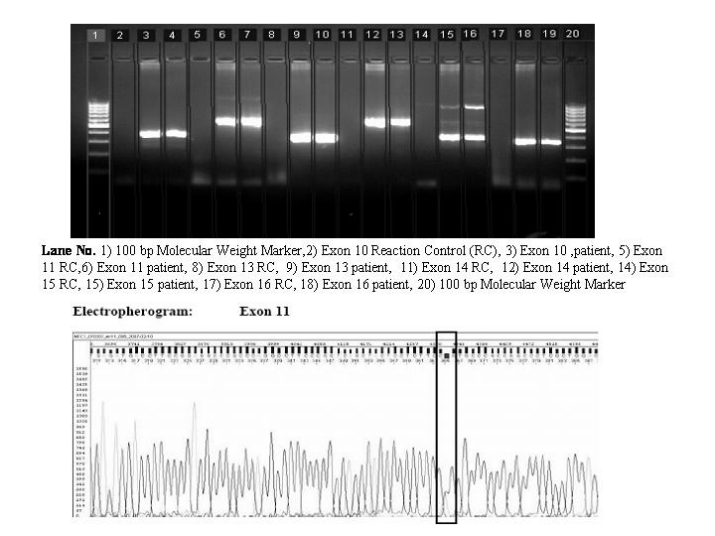
A. 2% Agarose gel electrophoresis of the index case. B. Sequencing Electropherogram of the Exon 11.

**Figure 4:**
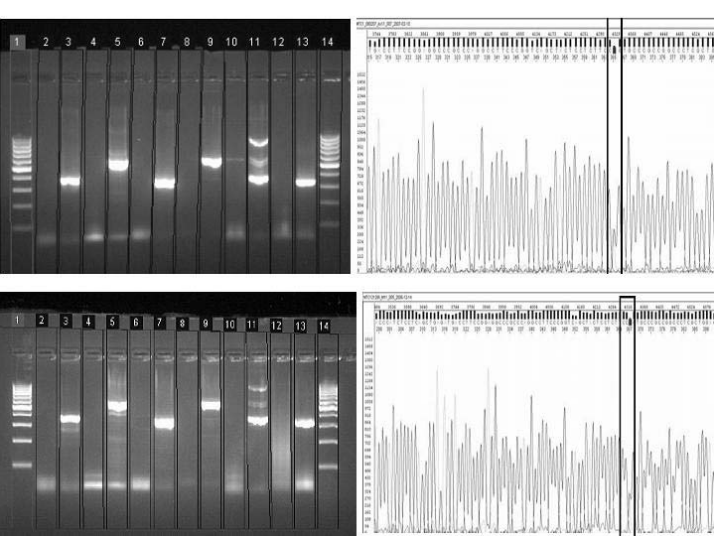
A. 2% Agarose gel electrophoresis of the daughter of the index case. B. Sequencing Electropherogram of the Exon 11 case. C. 2% Agarose gel electrophoresis of the grandson of the index case. D. Sequencing Electropherogram of the Exon 11.

**Figure 5:**
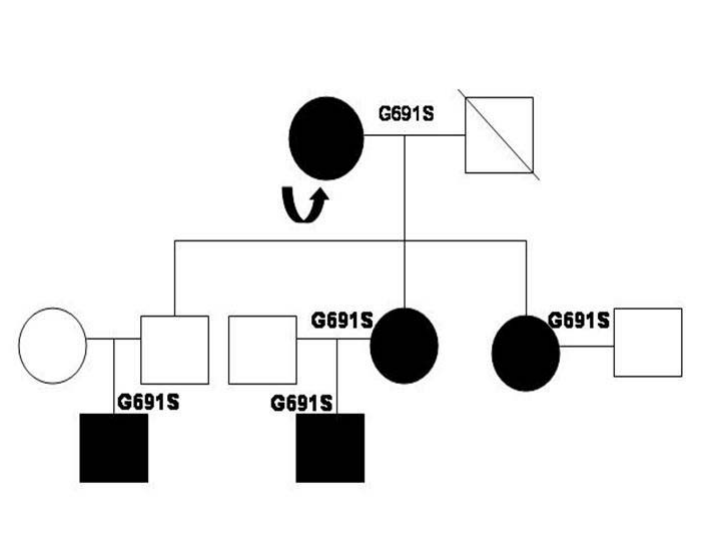
Pedigree of the family with the index case (arrow). Affected individuals (shaded dark) were heterozygous for the polymorphism.

The patients' DNA revealed a polymorphism in the heterozygous form in the *RET *Proto-oncogene at the exon 11 codon 691, resulting in GGT to AGT conversion, substituting Glycine to Serine (Figure 3A, B). Among her children, who underwent the similar genetic screening, both her daughters revealed the similar polymorphism in heterozygous form. However, the daughters' only child was spared with this mutation. In addition, one of her grandson also revealed the same polymorphism in heterozygous form (Figure 4A, B, C, D). Her son escaped the polymorphism (Figure 5). A follow-up of the patient, her daughters and grandson was recommended.

### Further treatment and follow-up

Subsequent to a total thyroidectomy with bilateral complete cervical nodal clearance, the patient underwent a diagnostic radioiodine scan with 100 μCurie that showed an uptake of 4.13%. Hence, a radio ablation with 195 milli Curie was performed after which the patient has been on a regular follow-up.

## Discussion

The occurrence of thyroid carcinomas with varying admixtures of MTC and differentiated carcinoma of the follicular epithelium has generated sufficient interest and controversy in terms of the histogenesis and appropriate designation for these tumors. In a comprehensive analysis, Guiu [14] has reviewed the available literature in terms of the histogenesis of a spectrum of such tumors. The present case of a collision tumor, including a MTC, PTC and a FVPTC was identified in a lady, who did not reveal any clinical manifestations an MEN syndrome. Histologically, discrete tumor nodules were identified in the left lobe and isthmus with features of MTC and PTC. Presence of amyloid stroma with strong calcitonin positivity confirmed the diagnosis of a MTC, whereas classical nuclear features with thyroglobulin positivity were helpful in confirming a PTC as well as a FVPTC. There was no intermingling of the two types of tumor cells, barring a diagnosis of a mixed thyroid carcinoma [3-8]. The lymph node deposits were of a MTC, unlike a mixed tumor, as noted earlier [9].

Lately, *RET *(rearrangements in tyrosine kinase) proto-oncogene has been implicated in the development of familial and sporadic MTC [15,16]. Several germline point mutations of this gene have been found to be affecting Exons 10, 11, 13, 14, 15 and 16 that have been linked with the development of MEN 2 and familial MTC. *RET *somatic point mutations and gene deletions have been identified in 40–50% cases of sporadic MTC [17]. Our case did not reveal a familial history of MTC. On genetic analysis, the patient displayed a unique G691S polymorphism that has been lately found to be associated with sporadic MTCs [18,19]. It is noteworthy that the high risk mutation was absent. It has been proposed that G691S polymorphisms might be important for its putative role in the pathogenesis of sporadic MTCs. This has been found to be associated with C-cell hyperplasia in the pretumor tissue of the differentiated radiation induced thyroid tumors [20]. As C-cell hyperplasia is considered as a preneoplastic lesion by some authors, there is a possibility that the G691S polymorphism induces C-cell hyperplasia. However, according to Elisei et al [18], it is difficult to deduce that normal subjects harboring G691S polymorphism are at a higher risk of MTC. Nevertheless, in a recent study by Robledo et al [19], it has been clearly suggested that the presence of G91S/S904S polymorphisms of the *RET *is related to the early appearance of symptoms in MEN 2A patients, and therefore, these polymorphisms could be considered as genetic modifiers. A G691S polymorphism might be included as a low penetrance gene that might be associated with a small to moderate increased risk for the development of a MTC. Although our index case was a lady, both her daughters and a grandson, displaying the similar polymorphism could be at risk for development of an early MEN2A syndrome.

The exact mechanism by which these polymorphisms affect the age of onset for MTC is unclear. It has been explained that the G691S variant occurs in the cytoplasmic tail of the RET amidst transmembrane region and the first tyrosine kinase domain close to the residue Y687 that was found phosphorylated in all MEN 2A cases, along with few cases of MEN 2B. Even though G691S is not considered as an oncogenic mutation, its functional role has been hypothesized. Higher levels of this polymorphism have been noted in sporadic cases of MTC vs normal subjects, thereby suggesting its role as a low penetrance gene. It has also been postulated that such variants are relatively common in the population may confer a much higher attributable risk in the general population than mutations in high penetrance cancer susceptibility genes [19].

Apart from a MTC, the present case also had discrete tumor nodules of PTC and FVPTC. In a literature review, Rossi et al [21] identified 21 such cases, including 3 cases from their own study that in addition, included genetic analysis. They noted C634A substitution in the exon 11 of the *RET *gene in the MTC component, while V600G *BRAF *mutation in the PTC tissues, thereby suggesting the possibility of discrete mutations in both the tumors. In the present case *BRAF *mutation analysis was not carried out.

Similar to ours, in their unique case of a collision tumor, Papi et al [11] identified a mutation on the *RET *gene. However, the affected Exon and codon was Exon 14 (codon 804). Recently, Melillo et al [22], while analyzing the transforming activity of RET mutations in PC CI3 follicular cells, in case of a family with affected siblings showing a collision tumor, postulated that RET point mutants behave as conditional oncogenes, able to predispose to PTC only under specific circumstances, such as, high level expression of the mutated allele in follicular cells. However, the present case displayed occurrence of a low penetrance gene. It is possible that low penetrance genes, acting over a long time, provide a 'fertile soil' for further mutations or differential expression, leading to development of collision tumors. It is suggested that occurrence of RET polymorphisms in such cases might validate the proposed hypotheses of 'field-effect' in cases of MTC, as well as about C-cells differentiating into follicular cells [13].

Therapeutically, it is important to identify whether cases of collision tumors should be given radioactive ^131^Iodine. In the present case, in view of an increased radioactive iodine uptake, post surgery, the patient was subjected to radio ablation. Identification of mutations or polymorphisms as G691S in the progeny of this case opens the possibility of discussing the screening in the subsequent family members. In such cases, calcitonin (CT) levels, especially post calcium or pentagastrin infusion have been found to be useful to decide upon therapeutic interventions as the polymorphism in itself is not like a high risk mutation. While basal calcitonin level is < 30 pg/ml, levels between 30 to100 indicate C-cell hyperplasia and above 100 pg/ml indicate presence of a MTC. CT levels between 30 to100 necessitate further investigations including USG/CT scan examination for the detection of thyroid nodule [23]. Presently, the patient's daughters have normal calcitonin levels and unremarkable thyroid. In view of unremarkable thyroids and normal calcitonin levels, therapeutic intervention has not been carried out in the patient's progeny. Nonetheless, they are on regular follow-up.

Even though screening modalities and therapeutic implications are known in cases of familial MTC, the same have not been established in sporadic MTCs. The present case of a collision tumor, displaying a unique G691 polymorphism, is suggestive of additional genetic modifiers of *RET *gene mutations in cases of sporadic medullary carcinoma thyroid. To our knowledge, such a polypmorphism has not been documented in any case of a collision thyroid tumor. Identification of such low penetrance genes provides a scope for genetic screening in cases of pure MTC, mixed or collision tumors. Documentation of more such cases, with a follow up is necessary to lay management guidelines for family members detected with the similar polymorphism, as of the index case.

## Competing interests

The author(s) declare that they have no competing interests.

## Authors' contributions

BR: Diagnosing pathologist, designed, prepared and drafted the manuscript

RRB: Resident Surgeon, procured the clinical details and collected the references

MAD: Resident pathologist, involved in the diagnosis and preparation of the manuscript

DC: Treating Oncosurgeon, provided the clinical details

AKD: Consultant Oncosurgery, supervised the clinical aspect of the manuscript

SA: Consultant Radiology, provided radiological inputs

SVK: Overall supervision and gave the final approval of the manuscript
